# New records of *Anopheles arabiensis *breeding on the Mount Kenya highlands indicate indigenous malaria transmission

**DOI:** 10.1186/1475-2875-5-17

**Published:** 2006-03-07

**Authors:** Hong Chen, Andrew K Githeko, Guofa Zhou, John I Githure, Guiyun Yan

**Affiliations:** 1 Program in Public Health, College of Health Sciences, University of California, Irvine, CA 92697, USA; 2 Kenya Medical Research Institute, Kisumu, Kenya; 3 International Centre of Insect Physiology and Ecology, Nairobi, Kenya

## Abstract

**Background:**

Malaria cases on the highlands west of Mount Kenya have been noticed since 10 – 20 years ago. It was not clear whether these cases were introduced from the nearby lowland or resulted from local transmission because of no record of vector mosquitoes on the highlands. Determination of presence and abundance of malaria vector is vital for effective control and epidemic risk assessment of malaria among both local residents and tourists.

**Methods:**

A survey on 31 aquatic sites for the malaria-vector mosquitoes was carried out along the primary road on the highlands around Mount Kenya and the nearby Mwea lowland during April 13 to June 28, 2005. Anopheline larvae were collected and reared into adults for morphological and molecular species identification. In addition, 31 families at three locations of the highlands were surveyed using a questionnaire about their history of malaria cases during the past five to 20 years.

**Results:**

Specimens of *Anopheles arabiensis *were molecularly identified in Karatina and Naro Moru on the highlands at elevations of 1,720 – 1,921 m above sea level. This species was also the only malaria vector found in the Mwea lowland. Malaria cases were recorded in the two highland locations in the past 10 years with a trend of increasing.

**Conclusion:**

Local malaria transmission on the Mount Kenya highlands is possible due to the presence of *An. arabiensis*. Land use pattern and land cover might be the key factors affecting the vector population dynamics and the highland malaria transmission in the region.

## Background

More than 3 million malaria cases, with one million deaths due to malaria, are reported in sub-Saharan Africa, each year [1]. Historically, no malaria case has been reported on the Mount Kenya highlands in central Kenya [2]. The residents on the highlands west of the mountain began to notice this disease about 10 years ago. Originally, it was believed that malaria was introduced from the Mwea lowland where most vehicular traffic passes through onto the highlands, and where the vector is *Anopheles arabiensis*. An alternative hypothesis was that a vector and parasite were introduced and malaria was transmitted locally on the highlands. However, no malaria-vector mosquito has so far been recorded on the Mount Kenya highlands [3,4], thereby arguing against this hypothesis. A third possibility was that the malaria was latent in the highlands until ecological and climatic changes modify the transmission patterns.

The emergence of malaria on the Mount Kenyan highlands is not only a health problem to the local residents who may have lower resistance to malaria parasite, but also poses a threat to tourists. Each year, between 10,000 and 15,000 tourists, from various parts of the world, come to the Mount Kenya National Park. Even more visit other parts of the mountain, and stay in the area for several days. Therefore, malaria is ranked as the first concern for travelers' health in those tourist areas [5].

The occurrence of malaria transmission appears to be strongly linked with the spatial distribution of vector mosquitoes, and hence to local ecology [6-9]. The identification of the vector species and concomitant distribution records are vital for effective malaria control. With species identification comes the associated knowledge of the biology of that species which, in turn, dictates appropriate control measures. In addition, information on the conditions that facilitate the survivorship and development of malaria vectors in an area previously free of them will be valuable to assess invasion potential of the vector species in other regions [9].

This study surveyed aquatic sites for the malaria-vector mosquito larvae and historical malaria cases on the Mount Kenya highlands and the nearby Mwea lowland. The results are valuable for malaria control in the context of effective vector management in central Kenya.

## Materials and methods

**Figure 1:**
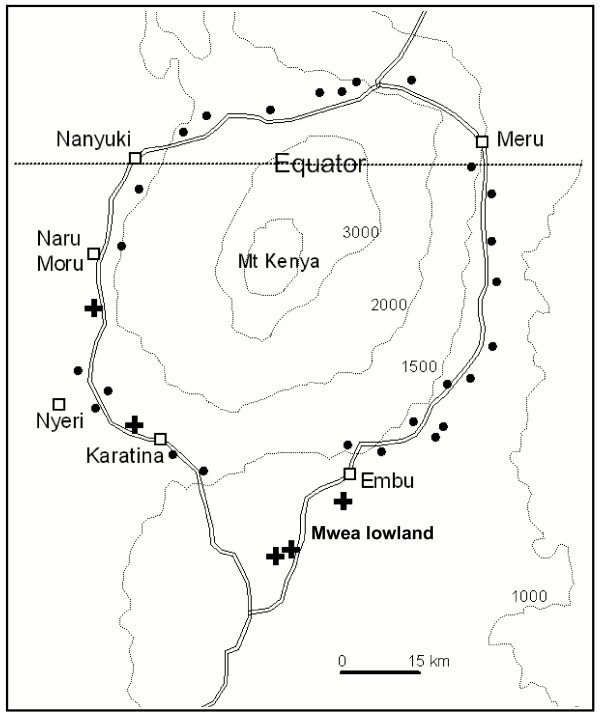
Map of aquatic sampling sites on Mt. Kenya highlands. + and ● along the primary road (double lines) indicate the positive and negative sites for *Anopheles arabiensis*, respectively. Dotted curves with numbers show the elevations (meters above sea level).

A survey of anopheline larvae was conducted at 28 aquatic sites on the highlands (> 1,400 m above sea level) around Mount Kenya and at 3 sites in the Mwea lowland adjacent to the highlands in the south during April 13 to June 28, 2005 (Figure 1). This sampling period was in a rainy season, which was favorable to detect breeding habitats. The surveyed sites were within a range of 3 km to the primary road surrounding the mountain, the belt where most highland residents live. A handheld GPS unit was used to record the coordinate and elevation at each site. The sites were mapped using ArcView 3.2^®^, a Geographic Information System (GIS). The collected larvae were reared into adults in an insectary at the International Centre of Insect Physiology and Ecology, Nairobi, for further morphological and molecular species identification [3,10]. The numbers of emerged mosquito adults from different sites were 300 from Karatina, 23 from Naro Moru, and hundreds from the three Mwea sites, respectively.

PCR species identification within *An. gambiae *complex was followed the procedures described by Scott *et al*. [10] and Chen *et al*. [11]. If a sample could not be identified after three PCR reactions, it was marked as unknown. Every anopheline mosquito collected on the highlands was screened by the PCR identification. For the lowland samples, randomly selected 30 specimens from each site were tested although hundreds of anopheline mosquitoes from each site emerged.

A questionnaire sheet [12] was used to record number of malaria cases and the age of the patients in a family on the highlands for the periods of past 0 – 5, 5 – 10 and 10 – 20 years. In each family two or three adults (37 – 65 year old) were interviewed on their malaria episodes, and records were made on the sheet by a researcher. In Karatina and Naro Moru areas, 11 and nine families were questionnaire-surveyed, respectively. In addition, 11 families were surveyed in Nanyuki area, further north to the former two areas.

## Results and discussion

Anopheline larvae were found only in five habitats: two on the highlands west to Mount Kenya and three in the Mwea lowland. At the site (1,720 m above sea level) 3 km northwest to Karatina on the highlands, 45 mosquitoes were identified as *An. arabiensis*. There were only two *An. arabiensis *mosquitoes detected at the site (1921 m) 13 km south to Naro Moru. At the three Mwea sites (1,140 – 1160 m), totally 85 mosquitoes, 25 – 30 per site, were identified as *An. arabiensis*. No *Anopheles gambiae *was found in this study. The presence of the *An. arabiensis *vector larvae at the five sites verified that *An. arabiensis *bred locally, indicating possible indigenous malaria transmission both on the highlands and in the lowlands.

Micro-climate and geography are decisive in vector distribution [4,9,13]. A high amount of rainfall around the mountain [5] provides sufficient water for the mosquito to breed. Topographically, the highlands on the eastern and western sides of Mount Kenya are quite different. The western highlands, where *An. arabiensis *larvae were found, are much more flatter than the eastern highlands. The western highlands have many still water sites such as ponds, puddles and swamps, and are less shadowed by trees than the eastern highlands. All these features are ideal for anopheline breeding habitats [13,14]. The breeding site south to Naru Moru was located at an elevation of 1921 m above sea level, and could be the highest breeding habitat for *An. arabiensis *ever recorded in Kenya [3,4,9]. The two sites with *An. arabiensis *larvae are close to the primary road, but it is unknown how and when *An. arabiensis *was introduced onto the western highlands. It needs to be clarified whether the mosquito breeds year round, or only in a short window period on the highlands with sufficient accumulated temperature for the malaria pariate, *Plasmodium falciparum*, to complete its sporogonic development [15,16]. On the other hand, the eastern Mount Kenya highlands are on deep slopes with running streams and covered by large forests, may contain fewer suitable aquatic sites for malaria-vector mosquitoes to breed.

In Karatina and Naro Moru areas where *An. arabiensis *was detected, the numbers of malaria cases recorded were eight in the past 5 years, three in the past 5 – 10 years and none in the past 10 – 20 years, indicating a trend of increasing malaria incidence over the past 10 years. Although the possibility of introduced patients can not be ruled out, these cases are more likely to be endemic because of the discovery of vector breeding on site. Moreover, among the eight malaria patients during the past five years, half were children. Since children are less likely to travel down to the lowland, the fact that there are cases of children with malaria in Karatina and Naro Moru, supports the hypotheses that there is indigenous transmission on the highlands west to Mount Kenya. Further north, in Nanyuki area, no malaria case was reported during this survey, which is concordant with no findings of *An. arabiensis *larvae there.

Change in land use patterns is an important factor effecting highland malaria transmission [6-8,13,17]. On the Mount Kenyan highlands, the population has increased at least 25% over the past 20 years. Population density in Karatina and Naro Moru areas is estimated at 530 persons per square km, much higher than those in the further north areas, e.g., only 81 persons per square km in Nanyuki [18]. With the increasing population on the highlands, enhanced human activities including deforestation, farming and livestock rearing could create more vector habitats. For example, at the site northwest to Karatina, hundreds of anopheline larvae were found in ditches holding spring water for crops in a field. The vectors emerging from this kind of habitat can play an important role in local malaria transmission.

The results of this study support the idea that local malaria transmission is on the western highlands of Mount Kenya. However, there are still many unknowns to the understanding of highland malaria. Parasitological tests should be done for the residents, especially among children, to verify the local transmission and its pattern there. The unknown mosquitoe adults and those dead larvae in this study need to be identified in the future to see if any other anopheline vector species exist in the research areas. Population genetic studies comparing the vectors and parasites, from the highlands and surrounding lowlands, may be used to infer whether those pests are native or newly introduced onto the highlands. If introduced, what are the possible sources? Studies to determine the level of highland people's resistance to the parasite may indicate if the residents have suffered from malaria for a long period. The model based on altitude data predicted a high epidemic risk, whereas the model using typical climate profiles forecast a low risk in Karatina and Naro Moru [2]. Refined models need to be developed based on geographic and micro-climatic data, and need to be tested with updated information on vector distribution and malaria transmission. The proposed studies on the vector, parasite and malaria cases are essential to controlling and preventing malaria for both residents and tourists.

## Authors' contributions

HC conducted field survey, PCR identification, data analyses and the manuscript draft. GZ assisted data analyses. AG, JG and GY supervised the study and assisted data analyses and manuscript preparations.
